# Oncogenic Hijacking of the PIN1 Signaling Network

**DOI:** 10.3389/fonc.2019.00094

**Published:** 2019-02-25

**Authors:** Alessandro Zannini, Alessandra Rustighi, Elena Campaner, Giannino Del Sal

**Affiliations:** ^1^National Laboratory CIB, Trieste, Italy; ^2^Department of Life Sciences, University of Trieste, Trieste, Italy; ^3^IFOM – Istituto FIRC Oncologia Molecolare, Milan, Italy

**Keywords:** signal transduction, tissue integrity, cancer target therapy, post-translational modification, prolyl-isomerase PIN1, organismal development, tumor development and progression

## Abstract

Cellular choices are determined by developmental and environmental stimuli through integrated signal transduction pathways. These critically depend on attainment of proper activation levels that in turn rely on post-translational modifications (PTMs) of single pathway members. Among these PTMs, post-phosphorylation prolyl-isomerization mediated by PIN1 represents a unique mechanism of spatial, temporal and quantitative control of signal transduction. Indeed PIN1 was shown to be crucial for determining activation levels of several pathways and biological outcomes downstream to a plethora of stimuli. Of note, studies performed in different model organisms and humans have shown that hormonal, nutrient, and oncogenic stimuli simultaneously affect both PIN1 activity and the pathways that depend on PIN1-mediated prolyl-isomerization, suggesting the existence of evolutionarily conserved molecular circuitries centered on this isomerase. This review focuses on molecular mechanisms and cellular processes like proliferation, metabolism, and stem cell fate, that are regulated by PIN1 in physiological conditions, discussing how these are subverted in and hijacked by cancer cells. Current status and open questions regarding the use of PIN1 as biomarker and target for cancer therapy as well as clinical development of PIN1 inhibitors are also addressed.

## Introduction

Cells respond to a continuous flow of intra- and extra-cellular stimuli through integrated signal transduction pathways. Within these signaling cascades proper levels, subcellular localization, complex formation, and activation timing of proteins are fundamental for an appropriate cellular response. Mechanistically, post-translational modifications (PTMs) of proteins underlie the dynamic molecular changes necessary to activate, transmit, and tune signals through the signal transduction network. PTMs impact on protein structure conferring new biochemical and biological properties. In this way, PTMs control several biological processes that require molecular switches, such as cell cycle progression, metabolic changes and cell fate decisions throughout organismal life (1).

**Figure 1:**
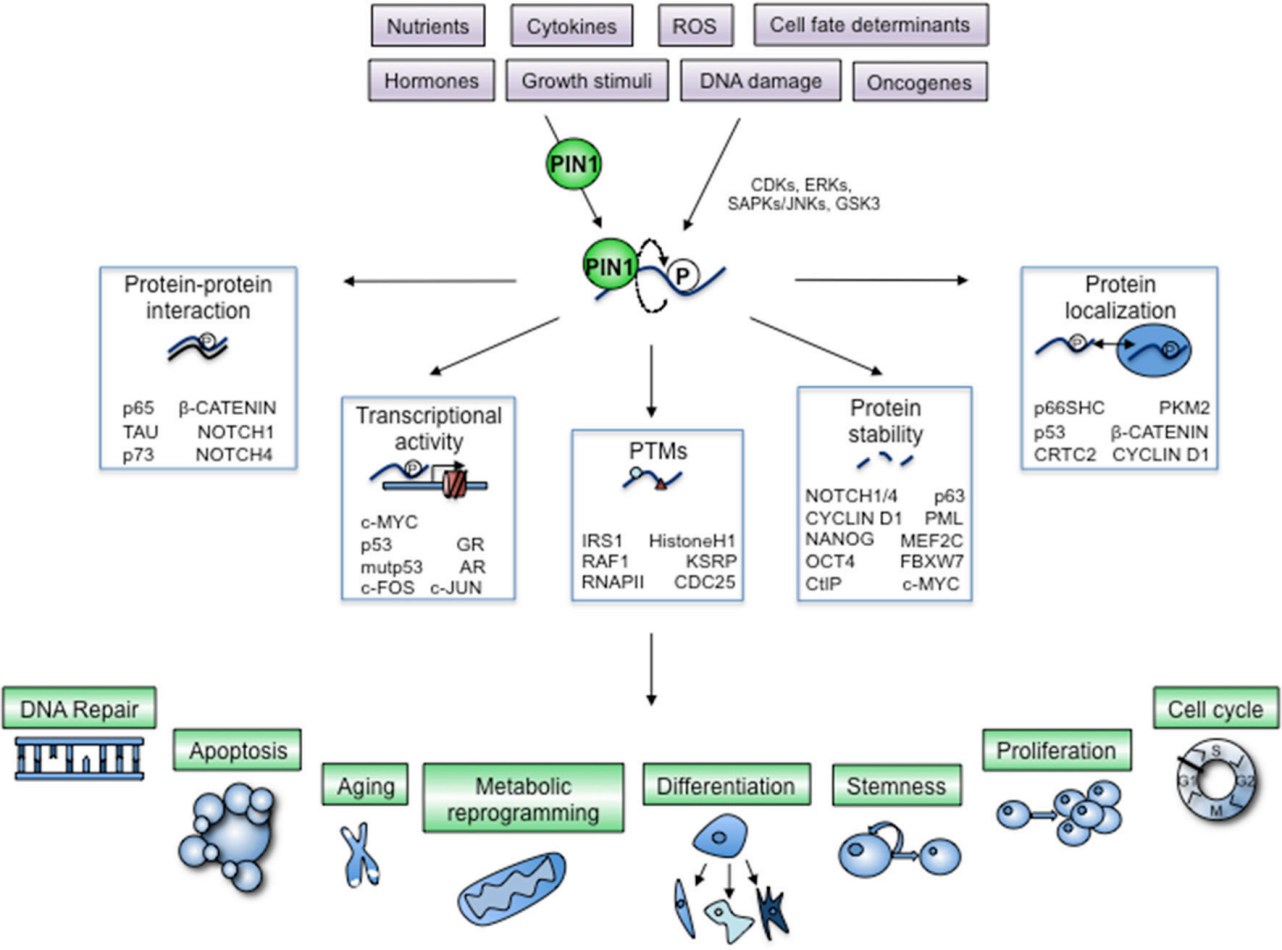
Regulation of cellular processes by PIN1-dependent modification of key cellular proteins. The prolyl-isomerase PIN1 is controlled by physiologic and pathologic cues and helps transducing phosphorylation signaling exerted by prolyl-directed kinases, often activated by the same stimuli (violet boxes). A growing list of cellular proteins are phosphorylated by these kinases and subsequently bound by PIN1, which induces a conformational change (bent arrow) with profound impact on their activation status by means of critical modifications of different biochemical and cellular properties (white boxes). By doing so, PIN1 adds a further layer of control to signaling pathways that are regulated by proline-directed phosphorylation. Proteins modified by PIN1 in fact can elicit cellular responses that involve multiple cellular and organismal processes, depending on the specific protein and on the cellular context (green boxes and blue drawings).

Phosphorylation of Serines or Threonines preceding a Proline (S/T-P) is one of the most frequent PTM occurring on a wide range of proteins of eukaryotic cells. This modification is tightly regulated by Proline-directed kinases and phosphatases devoted to their addition and removal, respectively (2). This PTM deserves particular attention, since Prolines adopt either a *cis* or a *trans* conformation, a slow but spontaneous structural change, that can elicit profound functional consequences. Peptidyl-prolyl *cis-trans* isomerases (PPIases) are the enzymes that accelerate the *cis-trans* rotation to a biologically relevant timescale. Among them, the prolyl-isomerase PIN1 uniquely recognizes S/T-P motifs when they become phosphorylated (pS/T-P). This substrate specificity is based on a conserved two-domain structure, where an N-terminal WW domain specifically recognizes pS/T-P sites (3) and a C-terminal PPIase domain performs the *cis-trans* isomerization (4). Hence, following Proline-directed phosphorylation, interaction with PIN1, and subsequent isomerization, the shape of target proteins undergoes changes that affect their stability, subcellular localization, protein-protein interaction, occurrence of other PTMs and ultimately activity (5). Accordingly, isomerization by PIN1 adds a new layer of control in signaling pathways that are regulated by phosphorylation, most notably the growth factor/RAS-MAPK, pRB/CDK/CYCLIN D1, p53, NOTCH, c-MYC, WNT/β-CATENIN, NF-kappaB, PI3K/AKT, and several other pathways (6–9). Through these signaling pathways PIN1 has been shown to impact several cellular processes, such as cell cycle progression, regulation of cellular metabolism and stem cell maintenance (Figure 1).

Both cell-autonomous and systemic effects of PIN1, through global regulation of phosphorylation-dependent events, are required for proper embryonic development and maintenance of tissue integrity in adulthood. As a consequence, subtle alterations of PIN1 expression or activity or of the phosphorylation status of its targets have been linked to a number of pathologies, ranging from inflammation to neurodegeneration and cancer. The observed alterations are likely the result of chronically compromised protein folding causing improper tuning or timing of relevant signaling pathways (5, 6, 8, 10–13).

PIN1 function is required for several biological hallmarks of cancer, as has been described in depth (7, 8, 10). PIN1 is overexpressed or hyperactivated in many types of cancers and its loss or inactivation blocks tumor growth. Indeed, *Pin1 knockout* in NOTCH3-dependent T-ALL (9), Eμ-myc lymphomas (14), or in MMTV-Ras/neu mammary tumors (15) in mice curbs tumorigenesis. Similarly, *knockdown* of *PIN1* in breast cancer xenografts was shown to curb tumor growth and metastasis formation and synergize with chemotherapy by dampening mutant p53 (mutp53) (16) and NOTCH1 (17) signaling, respectively. In addition, pharmacological inhibition of PIN1 dampens mammary tumor growth in a Myc/NeuNT mouse model (18) and metastasis development in a breast cancer xenograft (19). According to published results, PIN1 was shown to boost dozens of oncogenes or factors that promote proliferation, while inactivating several tumor suppressors (20), but which are the deranged underlying cellular processes is still incompletely understood.

**Figure 2:**
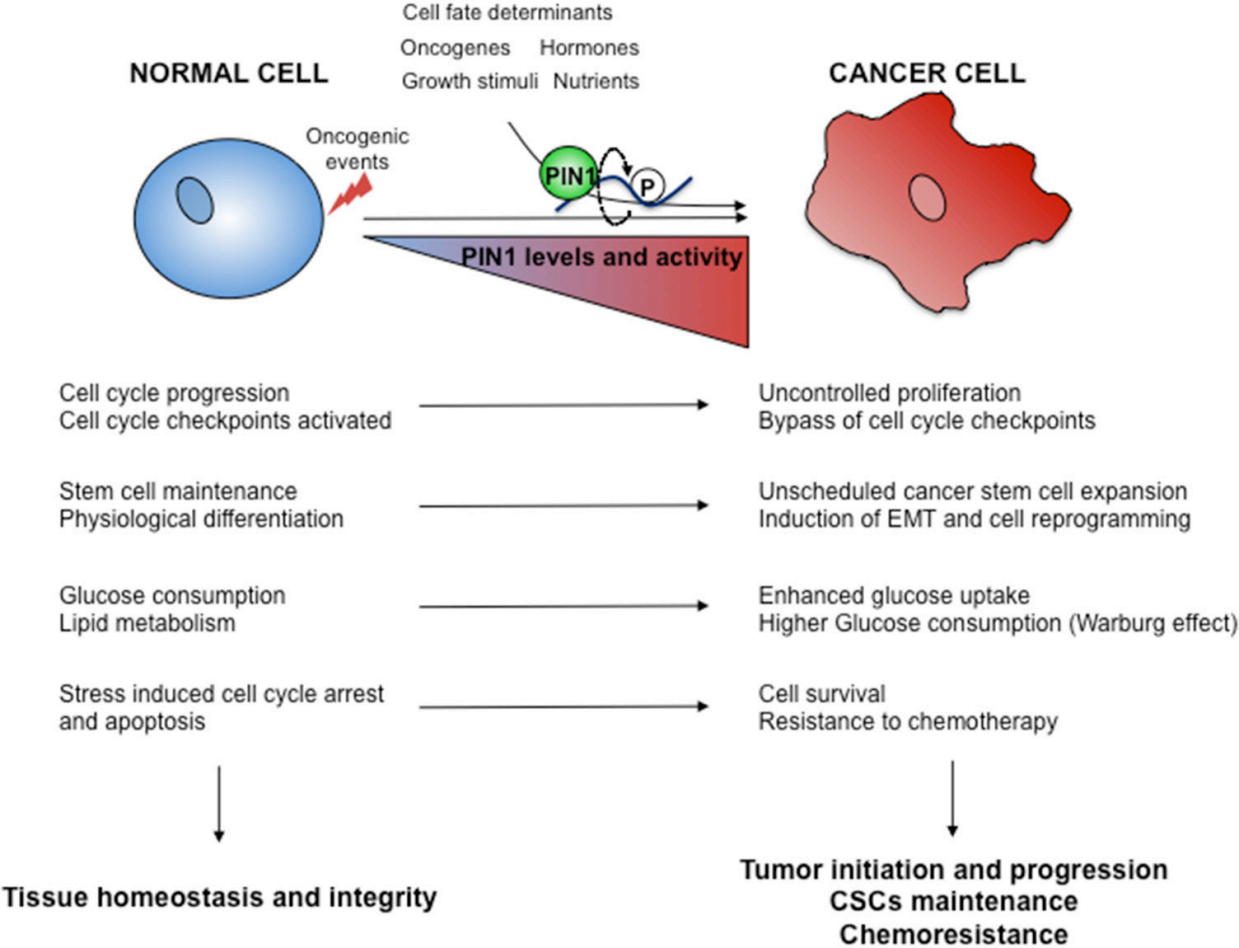
Oncogenic hijacking of the PIN1 signaling network. Oncogene activation and environmental cues cooperate in tumor development and progression by boosting PIN1 levels and activity as well as of its signaling networks. Several cellular processes regulated by PIN1 in normal cells **(left)** are thus subverted in cancer cells **(right)** to gain growth advantages.

In this review we will describe how cellular processes normally regulated by PIN1 could impact the pathogenesis of cancer. We will thus emphasize the relevance of PIN1 for cancer development and progression by orchestrating cellular processes that are emerging to be controlled by PIN1 in normal organismal development and that are hijacked in cancer, like metabolic reprogramming and response to cellular stresses, as well as dichotomous cell choices like stemness or differentiation, cell proliferation or cell death (Figure 2).

## Biological Roles of PIN1: Insights From Animal Models and Studies in Humans

PPIases are subdivided in three families, namely Cyclophilins, FK506 Binding Proteins (FKBPs) and Parvulins, predominantly defined on structural characteristics. The two former are large PPIases, also called immunophilins that are inhibited by immunosuppressants like Cyclosporin and FK506. The third family of Parvulins, to which PIN1 belongs, comprises smaller PPIases that do not bind to these drugs (21).

In yeast the PIN1 homolog ESS1 is the sole parvulin, while in mice and humans another Parvulin gene, encoding the proteins PAR14 and PAR17 (PAR14/17) (22) has been cloned. Cyclophilins, FKBPs, and PAR14/17 have specific substrate recognition motifs that differ from those of PIN1, nevertheless, these PPIases can share client proteins by targeting different portions on them (23).

More recently, within the human and mouse genomes, *Pin1*-like transcripts (*Pin1L*) have been found (24, 25). The human *PIN1L* encodes a putative protein of 100-amino-acids of which the N-terminal 63 amino acids are almost identical to PIN1 while it is fused to a different N-terminal 37-amino-acid tail. Instead, mouse *Pin1L* mRNA is widely expressed, showing highest levels in the testis and the protein shares 92% sequence identity with PIN1. Still it is not known whether these genes have a functional role or if they act as pseudogenes (24).

The biological functions of PIN1 have raised interest since its discovery in yeast (ScEss1p/Ptf1) (26, 27), where its deficiency leads to mitotic arrest and nuclear fragmentation (27). Since then, a number of *in vivo* loss-of-function and gain-of-function genetic models of PIN1 have been established and the biological function of *PIN1* orthologs has been characterized in different model organisms, including *Saccharomyces pombe* (*Pin1p*) (28), *Aspergillus nidulans* (*PinA*) (29), *Neurospora crassa* (*Ssp1*) (30), *Candida albicans* (*Ess1*) (31), *Drosophila melanogaster* (*Dodo*) (32, 33), *Danio Rerio* (*pin1*) (34), *Xenopus laevis* (*xPin1*) (35), and *Mus musculus* (*mPin1*) (36).

Studies in these model organisms indicate functional conservation of PIN1, mainly related to cell proliferation and metabolism. Moreover, while in unicellular organisms like *A. nidulans*, and *S. cervisiae* PIN1 is functionally essential, its ablation is associated with developmental defects in *C. albicans* (31), and metazoans like *X. laevis* (37), *D. melanogaster* (38) and *M. musculus* (36, 39–41). Indeed, lack of *Ess1* in *C. albicans* hampers cell growth and abrogates morphogenetic switching (31), while *dodo* serves for timely activation of the circadian rhythm (42) and its ablation impairs dorsoventral patterning of the follicular epithelium in the egg chamber in *D. melanogaster* (38). In *X. laevis* lack of *xPin1* impairs survival of embryos during development (35, 37). *Pin1 knock-out* (*Pin1KO*) mice display impaired neonatal motor activity (43), decreased fertility both in males and females (41), alteration of the mammary gland that fails to undergo proliferation during pregnancy, and decreased body weight (39). Moreover, these mice show premature aging phenotypes, such as testicular atrophy (39), alterations of bone homeostasis (44), retinal atrophy (39), and neurodegeneration (45). In addition, in aged mutant APP transgenic mice, *knock-out* of *Pin1* incites deposition of insoluble beta amyloids (46).

Human PIN1 was discovered by a two-hybrid screening aimed at identifying proteins that interact with NIMA-1, the key mitotic kinase of *Aspergillus nidulans* (47). In humans, deregulation of PIN1 levels or activity has been correlated to inflammation, obesity, diabetes, cancer, and neurodegeration (6, 48, 49). Indeed, neurodegeneration is mainly associated to low levels or activity of PIN1, while on the contrary overexpression of PIN1 is prevalently found in cancers and inflammatory diseases. Of note, the rs2233678 polymorphism in the human *PIN1* promoter is associated to decreased gene expression and to a lower risk of breast (50) and lung (51) cancer incidence, while the rs2287839 polymorphism hampers suppression of *PIN1* transcription by the AP4 transcription factor and correlates with delayed onset of Alzheimer's disease (52).

## Global Roles of PIN1 in Signal Transduction

The dynamicity of reversible phosphorylation of proteins represents a crucial step to transmit cellular inputs through the generation of integrating platforms for signal transduction (53). PIN1 acts on many cellular proteins and pathways in a phosphorylation-dependent manner, displaying a variety of functional consequences. Here we will describe the biochemical consequences of the structural modifications exerted by PIN1 on several proteins and their impact on global functions in signal transduction and cellular processes in different animal and human models.

### PIN1 Deciphers the Protein pS/T-P Code

Signal transduction by phosphorylation often involves sets of protein kinases (*writers*), phospho-binding targets (*readers*) and protein phosphatases that remove individual phosphorylations (*erasers*). Typically, the proteins that comprise this system are modular in nature harboring peptide domains with binding and/or catalytic properties (54). In this view, PIN1 might be considered as a “*reader”* in association with proline-directed kinases as “*writers,”* which include Cyclin-dependent protein kinases (CDKs), extracellular signal-regulated kinases (ERKs), stress-activated protein kinases/c-Jun N-terminal kinases (SAPKs/JNKs), p38 mitogen-activated protein kinases (MAPK), and glycogen synthase kinase 3 beta (GSK3β). Indeed, several reports have demonstrated that following proline-directed phosphorylation events on many proteins, PIN1 causes further modulation by inducing a conformational change and affecting their pattern of PTMs, for instance by favoring their association with other kinases, phosphatases, acetyl-transferases, etc. (55). In this regard, a biological interdependence has been found between PIN1 and the protein phosphatase 2A (PP2A), since PP2A is conformation-specific and effectively dephosphorylates only *trans* pS/T-P isomers (56). PIN1 and PP2A share many client proteins and show reciprocal genetic interactions (5). A paradigmatic example for such a PIN1-dependent sequence of PTMs is the priming phosphorylation of c-Myc by RAS/MAPK, followed by GSK3β phosphorylation and PIN1 binding, whose isomerization elicits the PP2A-mediated dephosphorylation and subsequent proteasome dependent degradation by the E3 ubiquitin-ligase FBXW7 (57). Other examples of PIN1-dependent regulation of PTMs at multiple levels are NOTCH1 (17, 58), RAF-1 (59), CYCLIN E (60, 61), KSRP (62, 63), TAU (56), and pRB (64, 65).

### PIN1 Optimizes Signal Transduction and Pathway Crosstalk

When a cell receives a signal, a physiological response is initiated by activating a string of biochemical reactions. Indeed signal transduction is accomplished by receptors, adaptors and amplifiers able to modulate a number of effectors like transcription factors that elicit a cellular response (1). Pathway activation can be achieved in many different ways, for instance by changing subcellular localization or enhancing the duration and quantity of the effectors. In this scenario, PIN1 fulfills the role of both fine-tuner and amplifier of signal transduction pathways by regulating single members of a pathway and also their cognate activators and inhibitors, finally determining the level of their activation state. The best described example concerns the growth factor activated RAS/MAPK pathway in human breast epithelial cells, since PIN1 was shown to act as an amplifier of this cascade at all the levels of signal transduction, including stabilization of the receptor (EGFR), enhancing activation of the transducing kinase (RAF-1), increasing the stability of the transcription factor (c-JUN) and of the effector (CYCLIN D1), while its ablation dampened this signal transduction with a consequent impairment of cell cycle progression (39, 59, 65–67).

**Figure 3:**
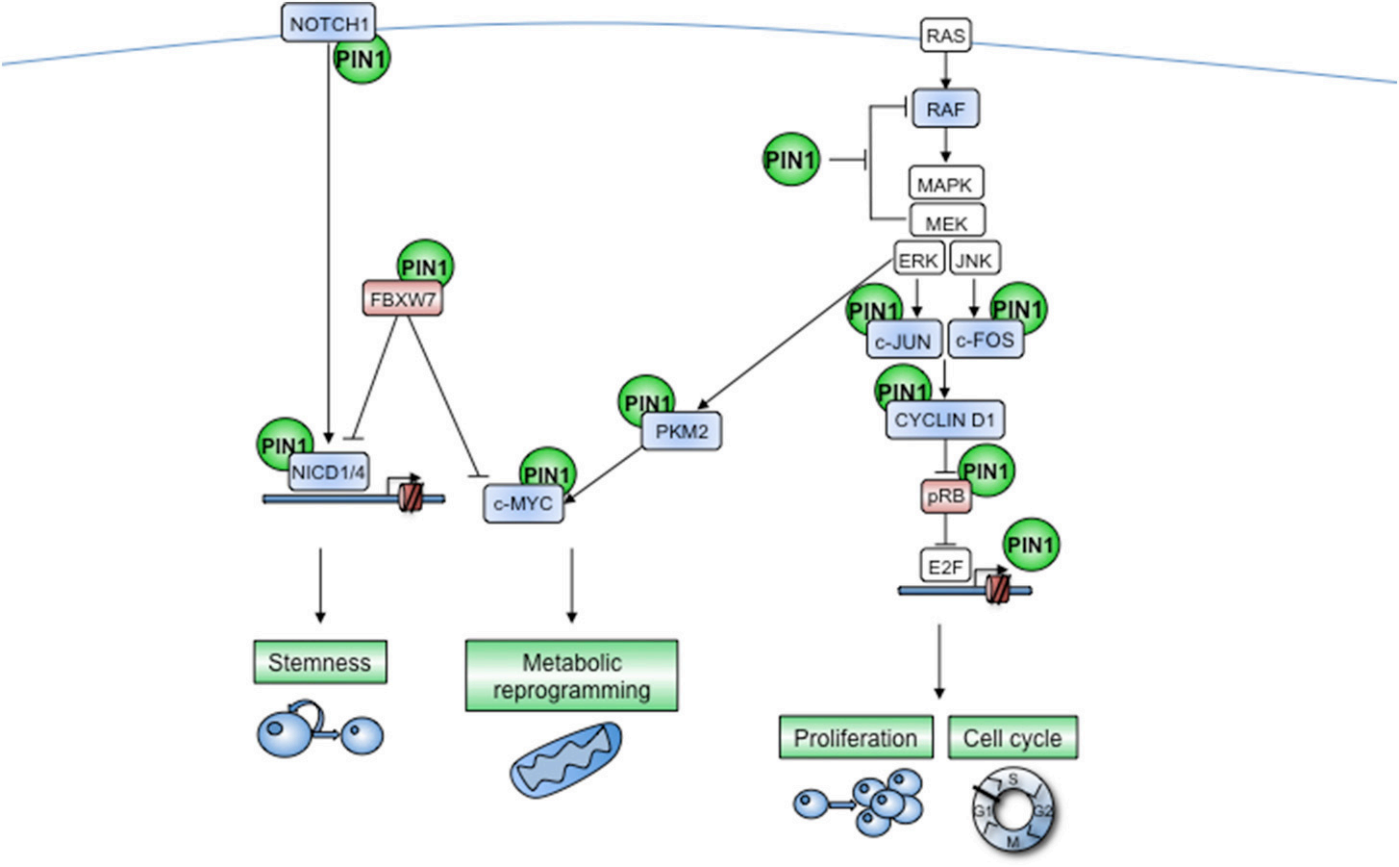
PIN1 acts at multiple levels of cooperating cellular pathways. NOTCH receptor and RAS-dependent pathways are indicated as examples of signal transduction pathways that are regulated by PIN1 at multiple levels. Furthermore, PIN1 enables active cross-talk between different pathways, e.g., by promoting nuclear translocation of PKM2 after EGFR-stimulated phosphorylation by ERKs, that in turn serves to enhance c-MYC transcriptional activity. **(Left)** The NOTCH pathway is activated by a ligand-dependent cleavage of the membrane-bound receptor that releases the Notch intracellular domain (NICD), which translocates into the nucleus where it promotes the transactivation of several target genes. This signal is turned off by the E3-ubiquitin ligase FBXW7 as a safeguard mechanism against excessive NOTCH signaling. PIN1 is harnessed by the NOTCH pathway to sustain its own signaling, indeed PIN1 boosts the cleavage of the NOTCH1 receptor and prevents the interaction of NICD1 and NICD4 with FBXW7, increasing in this way pathway activation, protein stability and transcriptional activity. In some contexts, PIN1 can also directly block FBXW7 activity. Moreover, some NOTCH transcriptional targets, such as CYCLIN D1 and c-MYC, are also direct PIN1 targets, thus suggesting that PIN1 may amplify also the NOTCH transcriptional program. Notably NICD1 and NICD4 directly promote Pin1 transcription. Blue color indicates PIN1-activated proteins, red color indicates PIN1-inhibited proteins. Arrows and blocked lines show positive and negative effects, respectively. **(Right)** In response to growth stimuli, RAS activates the Raf kinase, which in turn activates downstream MAPKs that phosphorylate c-FOS and c-JUN causing the formation of the AP-1 transcription factor that transactivates the CYCLIN D1 gene. CYCLIN D1-CDK4/6 complex, in turn, blocks pRb, unleashing the transcription factor E2F, boosting in this way cell cycle progression. PIN1 itself is a direct transcriptional target of E2F and strongly promotes the RAS/MAPKs/CYCLIN/CDK cascade at multiple levels: (i) by blocking negative feed-back regulation of RAF by MAPKs, (ii) by enhancing both transcription and protein stability of CYCLIN D1, and (iii) by blocking the activity of pRB.

As mentioned above, PIN1 activity is involved in many other signaling pathways (17, 43, 68–70) and controls the levels and activity of several kinases beyond those of the RAS/MAPK pathway as well as protein phosphatases (55, 56). Therefore, PIN1 could control not only the amplification of a single signaling cascade but also the cross-talk between these pathways, with important consequences for organismal development (Figure 3).

In the majority of cases, PIN1-induced conformational changes determine the direction and intensity of cellular responses and hence impact on cell fate by affecting the levels of key client proteins (5). Indeed, PIN1 controls the turnover of many substrates either favoring or blocking their recognition by E3 ubiquitin-ligases, enzymes that often require particular PTMs on their substrates (71). Moreover, PIN1 directly regulates the activity of the ubiquitin-ligase FBXW7 in particular cellular contexts (72), and, since PIN1 and FBXW7 in part share the same consensus on proteins, it can also antagonize FBXW7-mediated degradation of certain substrates. This occurs in the case of the intracellular domains of the NOTCH1 and NOTCH4 receptors, whose stability and transcriptional activity depend on the presence of these two enzymes. Moreover, in breast cancer cells with high levels of PIN1, NOTCH1 signaling is strongly activated despite presence of FBXW7, inciting expansion of breast cancer stem cells (CSC) and tumorigenesis (17) (Figure 3). The protein stability of key drivers of signaling pathways fundamental for organismal development and integrity, i.e., c-MYC (18), NANOG (73), OCT4 (74), p53 (6), p63 (75), β-CATENIN (68), relies on PIN1 levels and activity, thus strengthening the idea that PIN1 can impact cell signaling pathways by calibrating the amount of target proteins.

In addition PIN1 has been shown to affect the subcellular localization of many proteins (5). Nevertheless, the mechanism by which these proteins are shuttled as an effect of PIN1-dependent *cis/trans* isomerization is varied. While on one hand PIN1 can promote nuclear retention of CYCLIN D1 (39) and NF-kappaB (69) proteins, it induces a direct change in protein subcellular localization of some substrates, as in the case of Pyruvate Kinase 2 (PKM2), whose association with Importin alpha5 and its consequent nuclear translocation, a crucial step in the activation of the Warburg effect in cancer cells, is promoted by PIN1 (76). Conversely, PIN1 binds to and prevents the nuclear translocation of CREB-regulated transcriptional co-activator 2 (CRTC2) thus crippling transcription from cAMP-responsive elements, and ultimately fostering hyperglycemia in diabetic mice (77). Moreover, PIN1 is required for mitochondrial translocation of cytosolic phospho-glycero-kinase 1 (PGK-1) to promote the Warburg effect in tumor cells (78) and of the tumor suppressor p53, following stress-induced phosphorylation, to induce transcription-independent apoptosis (79). Thus, PIN1 activity is essential to determine the correct localization of client proteins within the cell at a given time.

### PIN1 Controls Gene Expression: From Transcription Factor-Specific to Global Gene Expression Mechanisms

**Figure 4:**
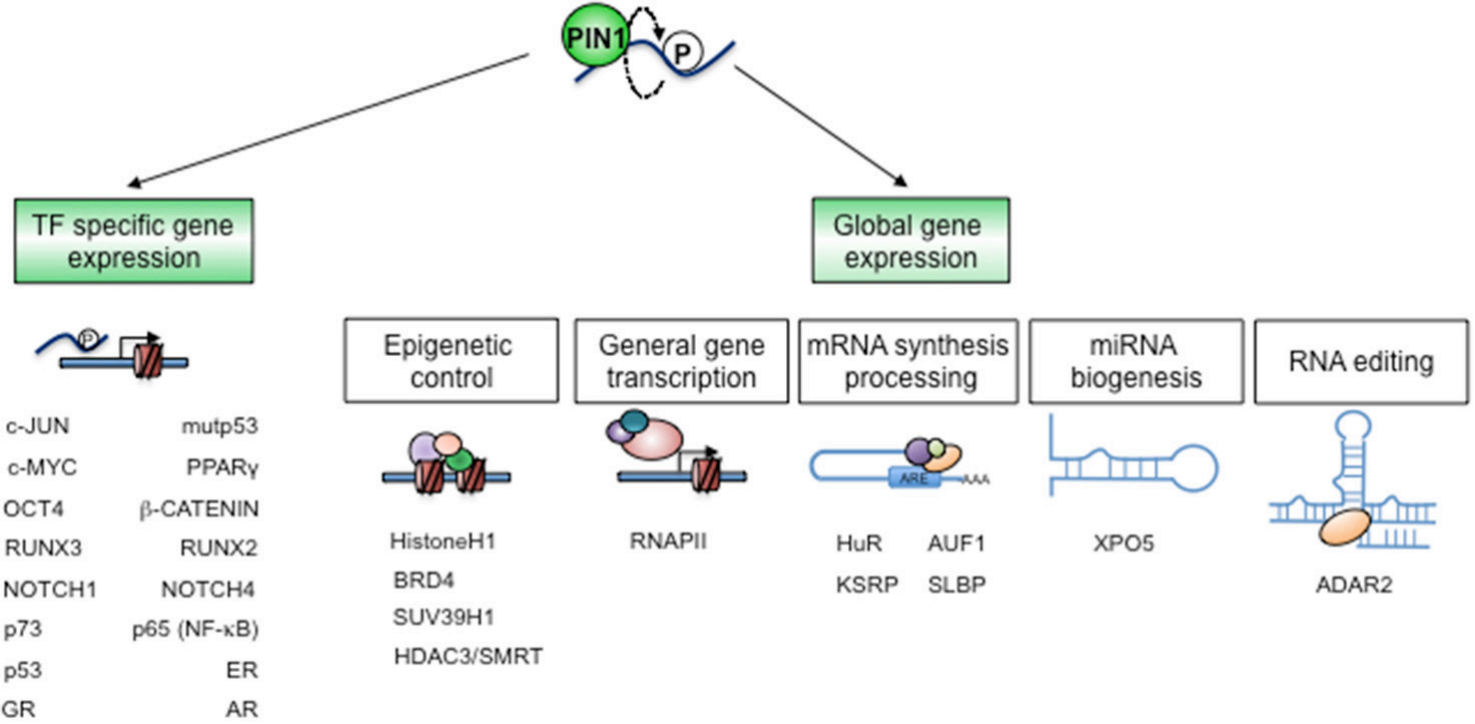
PIN1 controls gene expression. The establishment and dynamic regulation of specific cellular states largely depend on gene expression programs. PIN1 critically impacts these programs by direct structural modifications of specific TFs and co-factors **(left)**, as well as of chromatin and RNA regulators **(right)**, in a cell- and context-specific fashion.

A number of transcriptome profiles obtained in recent years from PIN1 silenced or *knock-out* cells demonstrated that this enzyme, although not a transcription factor (TF), has a sizeable impact on gene transcription (16, 18, 19, 62). Notably, PIN1 is able to impinge on gene transcription through the regulation of particular TFs and also by modulation of proteins recruited during mRNA synthesis and processing as well as factors governing chromatin accessibility (21) (Figure 4).

For several TFs, isomerization by PIN1 helps converting phosphorylation events in transcriptional outcomes (10, 80). In particular PIN1 can (i) modulate the association between TFs and co-activators, as described for Estrogen Receptor (ER) (81, 82), p53 (83), p73 (84), c-MYC (18), and several others (10), (ii) increase TFs protein stability thereby prolonging their transcriptional activity as described for NOTCH1 (17), and (iii) regulate the subcellular localization of TFs, allowing for their function, as shown for β-CATENIN (68).

An intriguing connection has been found between PIN1 and c-MYC. PIN1 binds to and controls the extent of c-MYC-dependent gene transcription in a bimodal way: on one hand it facilitates the loading of c-MYC to certain sets of genes, on the other hand it promotes its dephosphorylation and proteasomal-dependent degradation, thus tuning the timing of c-MYC transcriptional activity. In tumors, where c-MYC degradation is impaired, PIN1 promotes c-MYC-dependent gene transcription boosting the expression of genes involved in cell growth and metabolism (18, 57, 76). Interestingly, it has been shown that PIN1 is able to pre-anchor to un-phosphorylated c-MYC and the subsequent phosphorylation event increases the strength of this interaction (85).

Alteration of the epigenetic landscape is a hallmark of cancer. Epigenetic changes including DNA methylation, histone modifications, nucleosome positioning, and non-coding RNA expression, are responsible for cancer cell reprogramming (86). Knowledge about a role of PIN1 in controlling several epigenetic regulators in normal and pathological settings is growing and suggests a role for deregulated PIN1 in fostering cancer development also through epigenetic reprogramming of gene transcription.

In yeast, the HDAC subunits Rpd3 and Sap30 are targets of PIN1 that elicit repression of cell cycle genes: in this case they are negatively regulated by PIN1, and likely underlie the mitotic arrest of PIN1 mutants (87). A role for mammalian PIN1 in the control of HDAC family members has been unveiled in a study performed in human colon cancer cells treated with the HDAC inhibitor sulforaphane. It was proposed that, following treatment with this inhibitor, the nuclear HDAC3/SMRT co-repressor complex is phosphorylated by CK2 and the complex becomes dissociated and exported into the cytoplasm. It is hypothesized that, in this compartment, HDAC3 could interact with the phospho-serine or phospho-threonine binding cofactor 14-3-3 and shuttle back into the nucleus, but there is competition with cytoplasmic PIN1 that sequesters HDAC3, targeting it for degradation, thus hampering its gene silencing function (88).

In metastatic breast cancer cells, PIN1 overexpression controls histone H3K9 trimethylation (H3K9me3) through downregulation of the methyltransferase SUV39H1, fostering tumor growth. This result was validated in human breast cancer samples, showing an inverse correlation between PIN1 levels and those of SUV39H1 and H3K9me3. Importantly, reversal of the repressive chromatin state following PIN1 overexpression could elicit upregulation of CYCLIN D1 (89).

Another PIN1-dependent epigenetic mechanism in cancer cells occurs through stabilization of BRD4, a member of the bromodomain and extraterminal (BET) family of proteins that interact with acetylated histones and TFs. In human gastric cancer cells PIN1 induces a conformational change of phosphorylated BRD4 supporting its interaction with CDK9, thus improving BRD4-mediated gene expression relevant for gastric cancer cell proliferation and tumor development (90).

Phosphorylated histone H1 is found at sites of active transcription and its phosphorylation is required for induction of gene expression. Notably, PIN1 associates with phosphorylated Histone H1 at transcriptionally active sites and controls its turnover and binding to chromatin in order to reduce chromatin decondensation (91).

PIN1 also globally affects mRNA synthesis and processing. First, it has been shown that PIN1 directly impacts on gene transcription by assisting RNA polymerase II (RNAPII) function during the cell cycle where RNAPII preferentially associates with transcribed genes in S phase, while it dissociates from chromatin in a phosphorylation-dependent manner during M phase (92–94). Second, PIN1 affects the stability of mRNAs by acting on AU-rich element (ARE)-binding proteins HuR and AUF1, the KH-type splicing regulatory protein (KSRP), and the Stem–loop–binding protein (SLBP) (62, 63, 95). In particular, the abundance of many ARE-containing mRNAs with short half-lives, such as *GM-CSF, TGF-*β, and *PTH* mRNAs depend on PIN1 activity (62, 63, 96–98). Recent data also involve PIN1 in the regulation of microRNA biogenesis since it affects their nuclear-cytoplasmic shuttling in hepatocellular carcinoma (99).

Adenosine-to-inosine (A-to-I) RNA editing accounts for transcript diversification by modifying selected nucleotides in RNA molecules. Major enzymes responsible for this post-transcriptional modification are proteins of the ADAR family. One of these, ADAR2, was shown to be stabilized by PIN1 and to affect both transcript levels and permeability of the glutamate-gated ion channel receptor (GluR) (100). Later studies, investigating the landscape and regulation of editing in different tissues from a cohort of human and mouse samples revealed small effects of *Pin1KO* on global editing levels in a subset of mouse tissues like CNS, lung, spleen and small intestine (101).

All together these data clearly support a role for PIN1 in both gene-specific and global regulation of gene expression by modulating epigenetic regulators and transcription factors, as well as directly impacting the transcription machinery and post-transcriptional events. These data suggest that PIN1 could act as a determinant of cell identity by controlling the activity and expression programs of master TFs that define differentiation toward specific cellular lineages in normal cells (e.g., MEF2C for muscle cell identity, see section PIN1 Controls Stem Cell Reprogramming and Fate), while accumulating data argue for a potential role of PIN1 in supporting cancers that appear to be addicted to dysregulated transcriptional programs and elevated transcription rates, such as leukeamias and breast cancers (102). These tumors are frequently more sensitive to inhibition of the responsible transcriptional regulators than normal cells and thus these molecular circuitries represent interesting starting points for therapeutic interventions.

## Impact of PIN1-Mediated Prolyl-Isomerization on Cellular Processes Connected With Tissue Growth, Development and Cancer

### PIN1 Controls the Cell Cycle and Its Checkpoints

Like no other process cell cycle entry and progression, characterized by phosphorylation-dependent events, emphasize the multiple levels of intervention of PIN1. During the various steps of the cell cycle, the role of PIN1 in fine-tuning and regulating several phospho-proteins and thus the duration and intensity of various signals, is crucial to elicit cell cycle progression and cell division in response to pro-proliferative stimuli. Here we will only briefly review the role of PIN1 in key events of cell cycle and proliferation, and refer the reader to other reviews that have focused on this topic (7, 103).

While initial studies in yeast revealed a role for PIN1 in mitosis (27), further studies in mammalian cells unveiled that PIN1 dynamically controls cell cycle regulatory proteins and is required in all phases of the cell cycle (7, 10). PIN1 itself experiences moderate fluctuations of its levels and phosphorylation status in normal cells, which is critical to progress from one phase into the other: in particular CYCLIN D and E, whose levels are subjected to a rapid increase followed by degradation, strongly depend on PIN1 activity at both the transcriptional and post-transcriptional levels after phosphorylation by specific CDKs.

PIN1 controls the G1-S transition by directly repressing pRB. As a consequence pRB no longer inhibits E2F, and expression of CYCLIN D1 is induced (64, 65). Being also PIN1 an E2F target gene, its protein levels increase during the G1-S transition (67). The activation of CYCLIN D by PIN1 in turn further incites G1 phase CDKs to promote the transition from G1 to S-phase. PIN1 promotes CYCLIN E degradation and modulates p27^Kip1^ activity at a later stage (104–106), thus suggesting that PIN1 is controlling also the duration of the G1-S transition. Not surprisingly, *Pin1KO* MEFs exhibit defects in cell cycle re-entry after serum starvation (36). In S phase, PIN1 controls DNA and centrosome duplication (107) as well as the dynamic association of RNAPII on the promoters of transcribed genes (92). PIN1 also controls the G2-M transition, acting on the early mitotic inhibitor-1 (Emi1) and on the anaphase promoting complex (APC), thus coordinating the S and M phases (108). Moreover, PIN1 acts on CDC25C and WEE1 phosphatases, regulators of the CyclinB-CDC2 phosphorylation status (56, 109), thereby influencing entry in mitosis, and on separase and centrosomal proteins like CEP55 and Septin 9 during cytokinesis. All this evidence supports the idea that PIN1 is a fine tuner of cell cycle progression coordinating, in concert with cell cycle phase-specific CDKs and phosphatases, every phase of the cell cycle (103).

In addition, as shown in human, mouse, and zebrafish models, PIN1 participates in cell cycle checkpoints and promotes cell cycle arrest and/or apoptosis through activation of the tumor suppressor p53 (34, 79, 83, 110, 111) and its family member p73 (84), or by downregulating Che-1 (112) following genotoxic stress [for a comprehensive review see (6)]. Depending on the cell context and consistent with its role in boosting proliferation, PIN1 counteracts senescence (113, 114), an event that could be explained by the fact that PIN1 is able to regulate telomere length through TRF1 (115), cause inactivation of pRB (65), or target the senescence-inducing promyelocytic protein PML for degradation (116).

In human cancers PIN1 is prevalently overexpressed and essential for cancer cell proliferation and induction of centrosome amplification, two early clinico-pathological hallmarks of cancer (8). This has been observed in cell culture, in human tumors, and in *MMTV-PIN1* mice and shown to depend on the activity that PIN1 exerts on a plethora of cell cycle regulators, such as CYCLIN D, CYCLIN E, and c-MYC, just to cite some of the most relevant (8). The question arises as of how this can be reconciled with the function of PIN1 in inducing cell cycle arrest or apoptosis in cooperation with p53? As already mentioned (6–8), PIN1 can impact on the fate of cancer cells depending on the levels and activity of relevant client tumor suppressors and proto-oncogenes (7, 8, 20). Indeed, PIN1 may eliminate incipient cancer cells by cooperating with wild-type p53. Instead, as during tumor development p53 inactivation often occurs either by alteration of its pathway or by deletion of its gene (*TP53*), high levels of PIN1 might boost the activity of oncogenes. Even worse, *TP53* missense mutations that confer neomorphic oncogenic activities (117) are very frequent in human cancers and we have shown that PIN1 supports the oncogenic activities of these mutant p53 proteins in breast (16) and hepatocellular carcinomas (118).

### PIN1 Behaves as a Metabolic Adaptor

**Figure 5:**
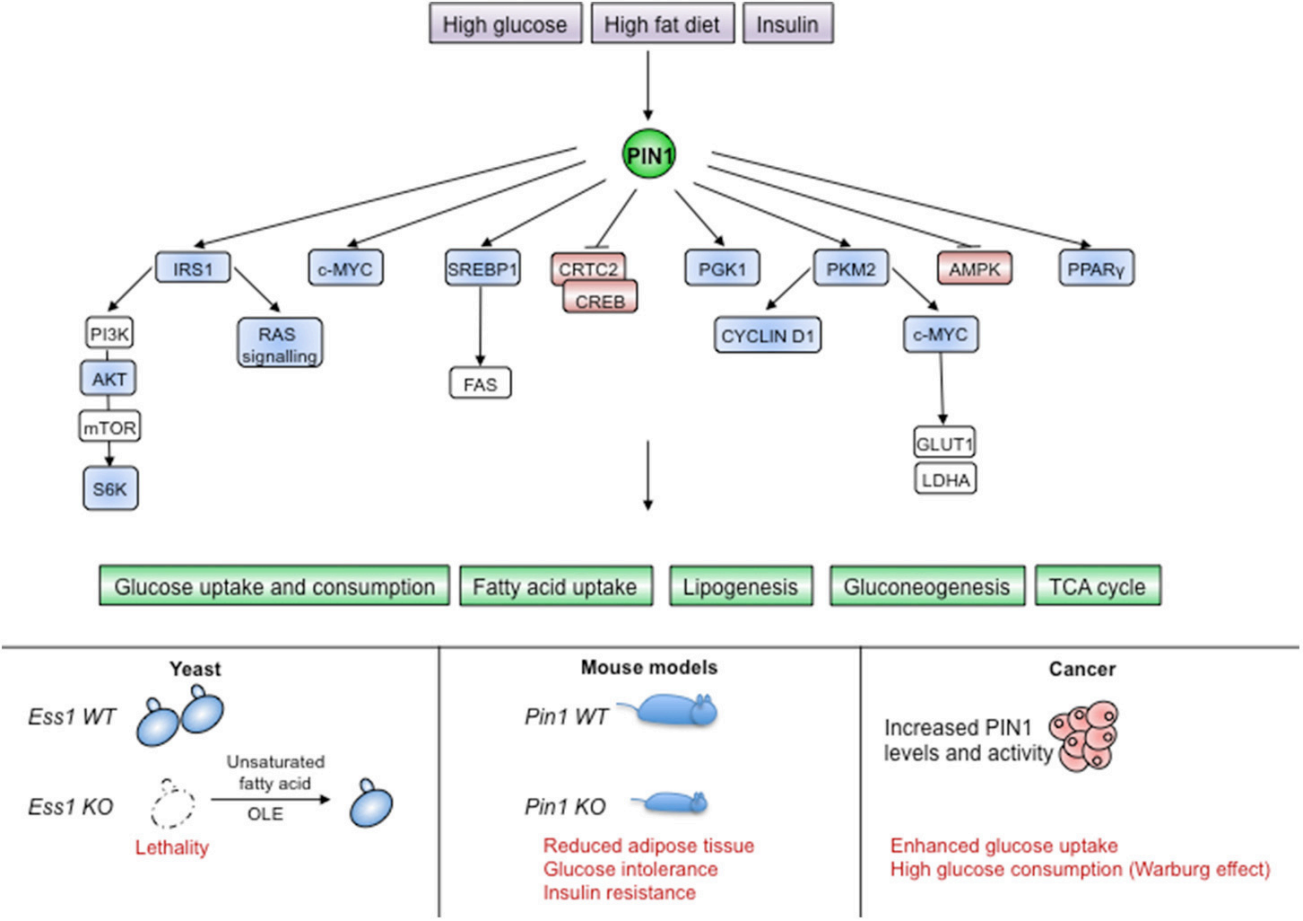
PIN1 is involved in sensing and transducing metabolic cues. **(Upper part)** Different nutritional and hormonal stimuli impinge on PIN1 levels and activity, which is necessary for the proper activation of metabolic pathways. **(Lower part)** Absence of PIN1 in unicellular organisms or metazoans causes lethality or metabolic alterations as shown for yeast and mice, respectively. Upregulation of PIN1 in human cancers supports the Warburg effect. Blue color indicates PIN1-activated proteins, red color indicates PIN1-inhibited proteins.

Proper cell growth, proliferation and survival depend on cellular metabolism (119). Old and recent findings have implicated PIN1 in the context of cell metabolism and metabolic diseases (48) (Figure 5). For instance in yeast, *Ess1* deletion causes a lethal phenotype that is rescued by overexpression of the delta-9-fatty-acid desaturase enzyme OLE1, or by supplying cells with unsaturated fatty acids. A key transcription factor of *Ole1* is the endoplasmatic reticulum (ER) membrane protein Spt23 that gets activated by regulated proteolysis of the C-terminal membrane anchor through the ER-associated degradation (ERAD) pathway following mono/oligo-ubiquitylation. The N-terminal, cleaved part of Spt23 then translocates into the nucleus to drive transcription of *Ole1*. *Ess1* finely tunes the ubiquitylation of Spt23, counteracting its poly-ubiquitylation and full degradation by ERAD, thus eliciting *Ole1* expression and exerting a protective function against lipotoxic stress (120).

The involvement of PIN1 in fatty acid metabolism seems to be conserved also in mammals, as PIN1 was shown to favor the transcriptional activity of the Serum Response Element Binding Protein 1c (SREBP1c), a master regulator of the fatty acid synthesis pathway and homolog of Spt23. In fact, in response to EGF signaling in breast cancer cells, PIN1 spurs SREBP1-dependent induction of the fatty acid synthase FAS, the rate-limiting enzyme of the fatty acid biosynthesis pathway (121). Another report showed that PIN1 had no effects on SREBP1 mRNA levels in mice (122), suggesting that it could act on SREBP proteins in correspondence of the ER membrane or of the transcriptional activity in the nucleus.

In mice it has been shown that PIN1 becomes upregulated in response to high glucose (123), insulin, and a high-fat diet (124, 125) and that it manages metabolic responses to diet and hormone stimulation (125, 126). Intriguingly, PIN1 augments the uptake of triglycerides and fosters differentiation of MEFs into adipose cells in response to insulin, whereas *PIN1KO* mice are insulin resistant, have glucose intolerance and display lower adipose tissue weight when fed with high fat diet (125). These reports suggest a role for PIN1 as metabolic adaptor. Accordingly, PIN1 is strongly intertwined with insulin signaling, as witnessed by its requirement in converting signals from insulin receptor (IR), Insulin-like Growth Factor-1 receptor (IGF-1R), and Insulin Receptor Substrate 1 (IRS-1) into intracellular activation of the RAS-MAPKs cascade and the PI3K-AKT-mTOR-S6K axis (127, 128). PIN1 promotes this signaling by fostering phosphorylation and activation of IRS-1 (125) and it is in turn transcriptionally upregulated by this pathway (70). In addition PIN1 has been shown to regulate proteins connected to insulin signaling such as PPARγ (129), AMPK (130, 131), and CRTC1-2 (77).

Most functional changes within a cell come along with an adapted metabolic switch. For instance quiescent and differentiated cells display a metabolism in which glucose is fully degraded to CO_2_ by glycolysis coupled to the Tri-Carboxylic-Acid Cycle. On the contrary, rapidly proliferating cells switch to aerobic glycolysis in which glucose is fermented to lactate even in the presence of oxygen (Warburg effect) (119). Of note, PIN1 crucially impacts EGFR-promoted Warburg effect in cancer cells by inciting nuclear localization of the Pyruvate Kinase 2 (PKM2), which induces c-MYC expression, resulting in turn in the upregulation of the glucose transport protein 1 (GLUT1) and lactate dehydrogenase A (LDHA) (76). Nuclear PKM2 causes transcription of several other genes, including CYCLIN D1, directly linking glycolytic metabolism with cell cycle progression. In turn, both c-MYC and CYCLIN D1 are central regulators of G1/S progression of the cell cycle, eliciting PIN1 expression via E2F transcription factors (67). Accordingly, PIN1 and c-MYC were found co-over-expressed in breast cancer, driving a gene expression pattern largely composed of genes involved in ribosome biogenesis/ translation and metabolism and enriched in poor-outcome breast cancer subtypes (18). Similarly, activation of ERK by hypoxia and oncogenic mutations can induce PGK1 phosphorylation, PIN1-mediated isomerization and consequent mitochondrial translocation to suppress metabolism of mitochondrial pyruvate further promoting the Warburg effect (78).

It seems that the general action of PIN1 downstream of glucose, insulin, fat or other growth factors that cause a metabolic switch, is to improve glucose uptake, glycolysis and lipogenesis. This is relevant, since metabolic pathways are not only engaged as a biochemical response to diet or energy demand, but also as mechanisms by which cells adapt to different stimuli and control their fate (132). In this view, we might speculate that metabolic reprogramming by PIN1 could be determinant for its pro-oncogenic effects. Indeed, reconsidering older studies where lack of PIN1 was shown to curb tumor growth induced by oncogenes such as RAS, HER2 (15), or NOTCH1 (17, 58), we might speculate that this might result not from a block of proliferation *per se*, but rather by prior restriction of the glycolytic pathway.

### PIN1 Controls Stem Cell Reprogramming and Fate

Organismal development and maintenance of tissue integrity are guaranteed by a correct balance between differentiation and stemness and by a proper determination of cell identity (133, 134).

**Figure 6:**
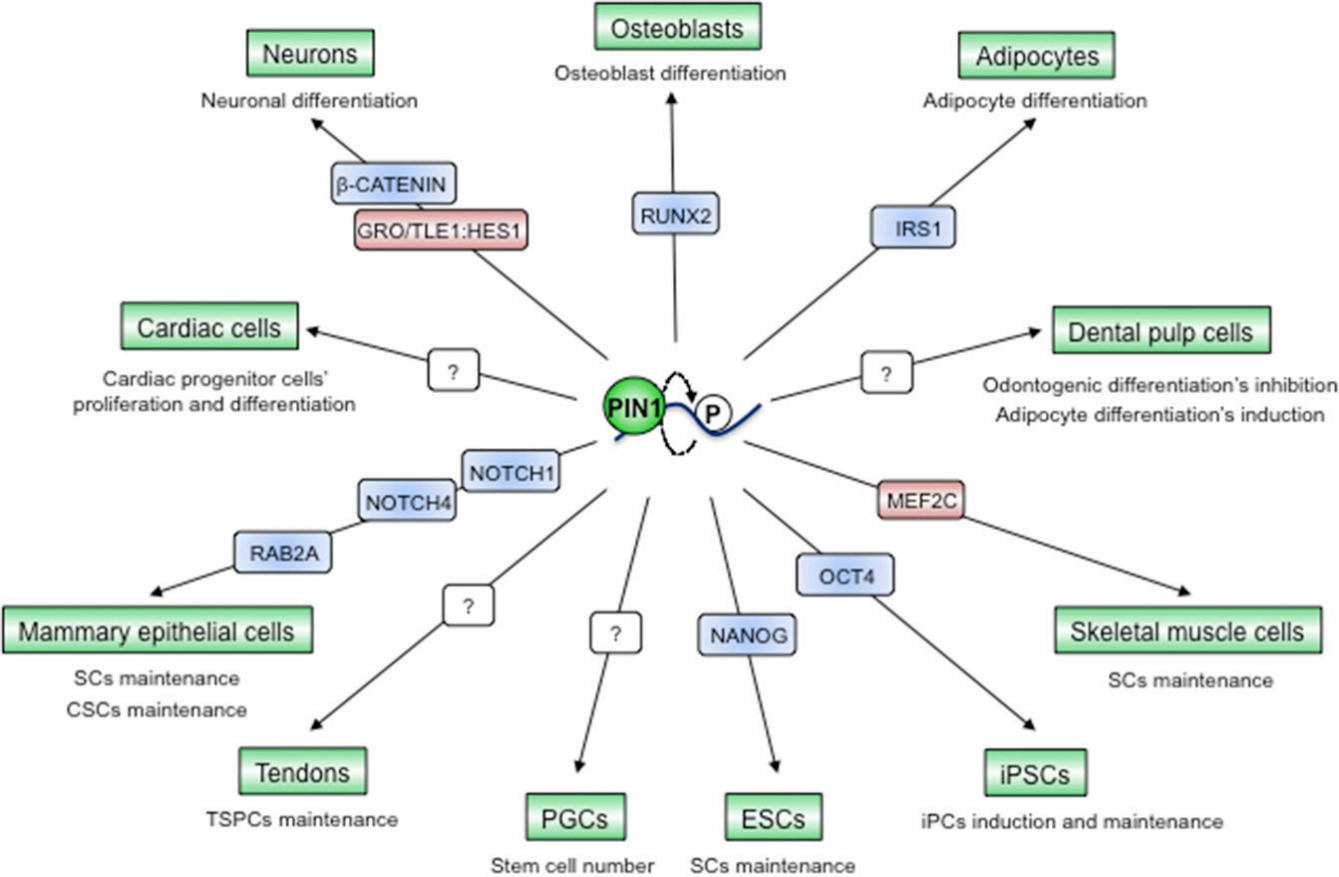
PIN1-dependent prolyl-isomerization serves as a pivotal hub to preserve tissue integrity and to maintain cancers' regenerative potential. PIN1 has been described as a fundamental element for the determination of cell fates in different tissues through interaction with and modulation of different proteins, mainly TFs. Blue color indicates PIN1-activated proteins, red color indicates PIN1-inhibited proteins, question marks indicate unknown protein substrates of PIN1.

High levels of PIN1 were shown to be relevant for the maintenance of pluripotency of mouse embryonic stem cells (ESCs) by upregulating the stemness factor NANOG (73). Later on it was shown that PIN1 is indispensable for the induction and maintenance of pluripotency in induced pluripotent stem cells (iPS) by interacting with phosphorylated OCT4, preventing its ubiquitination, boosting its protein levels and activity. Accordingly, PIN1 levels are higher in iPS and ablation of its activity suppresses colony formation (74) (Figure 6).

The ability of PIN1 to control stemness features seems to be conserved also in stem cell niches of the adult. Indeed, the defects in many tissue compartments observed in *Pin1KO* mice could be partially explained by a role of PIN1 in regulating the balance between stemness and differentiation. For instance, PIN1 serves to maintain the numbers of primordial germ cells (PGCs) and, as a consequence, *Pin1KO* mice have impaired fertility (41). In addition, in the developing mouse cortex PIN1 promotes the neurogenic fate through repression of GRO/TLE1:HES1-mediated inhibition of neuronal differentiation on one hand (135) and through upregulation of β-CATENIN in neuronal progenitors on the other (43). On the contrary, PIN1 preserves muscle stem cells by targeting the MEF2C myogenic transcription factor for proteasomal degradation in the nucleus, while the nuclear-cytoplasmic shuttling of PIN1 triggers skeletal muscle cells' differentiation (136). Similar scenarios have been observed also in cardiac progenitor cells (114), adipocytes (126), osteoblasts (137, 138), dental pulp cells (139), and mammary stem cells (17, 140, 141) (Figure 6). All together these data raise the question as of whether the phenotypes observed in adult *Pin1KO* mice could derive also from impaired stem cell potency and a lower regenerative potential of particular tissues.

Like in the normal mammary gland, PIN1 acts as a fundamental regulator of stem cell features also in cancer stem cells (CSCs) of this tissue compartment. Indeed PIN1 critically controls CSC self-renewal, replicative potential and frequency and these cells are strongly addicted to high PIN1 levels and activity, as demonstrated in both human and mouse tumors (17, 140, 141). PIN1 was shown to regulate breast CSCs' expansion through regulation of the NOTCH1/4 and Rab2A GTPase pathways (17, 141).

A growing body of evidence highlighted that PIN1 can globally affect cellular plasticity and reprogramming by acting upstream and downstream to regulators of epithelial-to-mesenchymal transition (EMT) (17, 140, 142), an embryonic process that is hijacked during cancer progression. Absence of epithelial constraints underlies this phenomenon that preempts the detachment of invasive cancer cells from the primary tumor. High levels of PIN1 were shown to induce EMT and causing E-cadherin downregulation and Vimentin upregulation in both normal and cancerous breast epithelial cells (17, 66, 140) by activating Snail or Stat3 that act via PTEN-PI3K/AKT, NF-kappaB, NOTCH1/4 (17, 143), or oncostatin M (144), respectively.

All these results clearly indicate that PIN1 can be considered as a cell-fate determinant both in normal and in neoplastic contexts and that alterations of its levels, activity or sub-cellular localization could compromise the integrity of many tissues or incite neoplastic proclivity.

### PIN1 Contributes to Orchestrate Cellular Responses to Cell-Intrinsic and -Extrinsic Cues

Cells exposed to bioactive stimuli and metabolites from inwards the cell or from the environment, such as growth factors, hormones, and xenobiotics, undergo adaptive responses to properly direct their fate and to safeguard cell and tissue integrity. PIN1 has a central role in tuning the ability of cells to both sense and transduce various stimuli and to elicit integrated cellular responses, thus determining cell fates during dynamic biological processes. While in normal conditions PIN1 activity ensures a homeostatic equilibrium, it appears that during pathological processes, such as inflammation and cancer, deregulated PIN1 exacerbates the diseased state.

For example PIN1 tunes the abundance and activity of both growth factor and hormone nuclear receptors and their molecular networks (10). Most notable examples are EGF and estrogen receptors (EGFR and ER) whose signaling cascades require the activity of PIN1. In fact, PIN1 stabilizes EGFR proteins and activates downstream kinases, such as MAPKs, PI3K-AKT, and CDK2 following growth factor stimulation. These kinases in turn phosphorylate ER, its co-repressor SMRT and its co-activator SRC-3 and elicit their interaction with PIN1. SMRT is subsequently targeted for degradation, while SRC-3 is activated, enhancing ultimately ER-dependent gene transcription (81). This cross-talk appears to be relevant for morphogenetic processes such as expansion of the mammary gland during pregnancy, while in breast cancers with high levels of PIN1, it confers resistance to selective estrogen receptor modulators, such as tamoxifen (81). PIN1 acts as a coactivator of several other hormone or growth factor receptor signaling pathways, ranging from Progesterone receptor to parathyroid hormone synthesis (63, 80, 145–147). Further investigation of the developmental consequences of such interactions is needed.

Integrated responses are required also following genotoxic stress. This is particularly relevant for DNA damage induction following hyperproliferative stimuli in pre-cancerous settings or by means of radio- and chemotherapies in tumor cells. Depending on the stimulus, the entity and the cell type, DNA damage can elicit cell cycle arrest, DNA repair, or apoptosis. As mentioned above, PIN1 coordinates all these processes by orchestrating both upstream and downstream functions of several factors, including ATR (148), p53, and p73 (6). For instance, PIN1 activity helps displacing negative regulators, such as Mdm2 (110, 111) and iASPP (83, 149), while activating upstream kinases, such as HIPK2 (79), to promote p53 nuclear and mitochondrial pro-apoptotic functions, respectively. Interestingly, downstream of chemotherapeutic treatments that induce double strand breaks (DSBs), PIN1 influences the choice of DNA DSB repair mechanisms. In particular high PIN1 levels promote non-homologous end-joining (NHEJ) at the expense of homology-directed recombination (HR), directly impinging on DNA ends resection by interacting with CtIP following its phosphorylation by CDK2 (150). Given that p53 regulates HR, the choice of the DSB repair pathway in cancer cells with high PIN1 levels may thus depend on whether p53 function is still present or lost (150).

Apart from its action in managing the consequences of cell damage induced by chemotherapeutics, in tumors PIN1 strongly correlates with chemoresistance, a major problem for the eradication of tumors and an unresolved cause of death. Indeed, we found that PIN1 inhibition in breast cancer xenografted mice sensitized tumors cells to paclitaxel treatment, not only by reducing the tumor mass but also by preventing the enrichment of the CSC population, a mechanism known to spur tumor recurrence after therapy (17). Such strong effects can be explained by considering the key role of PIN1 in boosting CSCs number, EMT features and the protein levels of factors involved in chemoresistance (NOTCH1, NOTCH4), pro-survival (BIRC-5, MCL-1), and detoxification (ABCG2) (17, 58, 151). In addition, PIN1 confers resistance to taxanes by reducing the activity of the Hippo pathway kinase LATS2, following CDK1 phophorylation (152).

An intriguing interplay between PIN1 and oxidative stress has emerged since the discovery that PIN1 is able to potentiate the mitochondrial accumulation of the lifespan regulator p66^shc^, thus inducing apoptosis in response to ROS (153). In accord with this finding, in human aortic endothelial cells exposed to high glucose, PIN1 is upregulated and promotes p66^shc^ mitochondrial translocation, reduction of nitric oxide and activation of NF-kappaB dependent inflammation. Consequently, *Pin1KO* mice are protected against diabetes-induced mitochondrial oxidative stress, endothelial dysfunction, and vascular inflammation (123). In another publication *Pin1KO* MEFs were shown to resist to H_2_O_2_-induced cell death because of upregulation of phosphatidylinositol-5-phosphate (PtdIns5P) (154). The impact of PIN1 on the oxidative stress response is still poorly characterized and seems to be context and tissue dependent, and thus deserves further studies. Nevertheless, given that tumor progression depends on oxidative stress (155), the question arises as to whether PIN1 pro-oxidant activity could foster tumor growth and aggressiveness.

## Regulation of Pin1 Expression, Subcellular Localization and Activity

Under physiological conditions, PIN1 levels and function are subjected to tight transcriptional and post-transcriptional regulation, at variance with other PPIases that are mainly constitutively expressed (156). First, scattered information about PIN1 expression in different organisms, such as mouse and zebrafish, show that PIN1 is prevalently expressed in embryonic and proliferating tissues (39), except for the central nervous system, in which PIN1 expression is induced following neuronal differentiation (34, 43, 135, 157). Moreover, in zebrafish embryos it was clearly demonstrated that mRNA and protein levels do not always correspond and, while established cell lines have prevalently nuclear PIN1, primary cell cultures or tissues often display cytoplasmic re-localization of PIN1 (see Ibarra et al. (34) and references therein). These and other studies suggest that in different developmental stages and tissues, PIN1 can be regulated not only at the level of mRNA expression, but also at those of mRNA stability and processing, as well as by critical PTMs and localization to specific cellular microdomains, that ensure proper adaptive cellular processes. Notably, while PIN1 function seems to be dispensable during development and adulthood of higher metazoans, its levels and activity enhance and become crucial for stress-induced cellular responses *in vivo*, such as during ionizing radiation in zebrafish (158), high glycemia (123), and high fat diet in mice (124).

The above-mentioned observations hold true also in conditions of tumor growth in mice and human models. Indeed, in spite of a very low mutation frequency of the *PIN1* gene (8), in human cancers PIN1 is prevalently overexpressed (159). High *PIN1* mRNA expression is strongly associated with cell proliferation. Indeed, *PIN1* is transcriptionally regulated by E2F in response to growth factors (67, 70) and by NOTCH1/4 activation in cancer (17, 58). On the contrary, the *PIN1* rs2233678 (−842G>C) promoter polymorphism is associated to a reduced expression of the gene and a reduced risk of cancer (50, 51). *PIN1* is also negatively controlled by tumor-suppressor microRNAs, such as miR-200b (160), a promoter of anoikis, miR-200c (140) that restrains EMT in breast cancer, and by miR-296-5p in prostate cancer (161), and some others only recently identified (20). In addition to PIN1 transcription, the binding and enzymatic activities of PIN1, its cellular localization and turnover are also finely regulated at the post-translational level, mainly through phosphorylation, SUMOylation, and oxidation (162–170).

All these data clearly show that PIN1 levels and activity are regulated at several levels that can be deranged during oncogenesis and in turn have positive repercussions on oncogenic signaling and tumor phenotypes (Figure 2).

## PIN1 Targeted Therapies to Treat Cancer

Current challenges for cancer therapy regard the possibility to simultaneously target multiple deregulated pathways that are causative for cancer progression and to effectively hit CSCs, while having none or acceptable side effects. PIN1 seems to be an ideal target that could conciliate all these requirements. Indeed, first of all, PIN1 is a unique enzyme that is overexpressed in many tumors. Second, *Pin1KO* or pharmacological inhibition drastically impairs tumor growth and dissemination (8, 19). Third, *Pin1KO* mice are viable and show only marginal defects. Such robust cellular effects on tumor growth and development that arise from loss of PIN1 function, while having milder consequences on normal tissues, are likely due to the modest effect on many members of the same oncogenic signal transduction pathways, rather than from a stark effect on one single client protein. Fourth, PIN1 has been shown to boost the CSC population in breast cancers and it represents a functional node for the cooperation between oncogenic circuitries, reason why tumors, but not normal tissues, are addicted to PIN1. Fifth, PIN1 knockdown or pharmacological inhibition sensitizes cancer cells of different origins to chemo- and target therapy, such as breast cancers to 5-fluorouracil, Taxol (151), and rapamycin (171), hepatocellular carcinoma to sorafenib (172), colon cancer to Taxol (72) and acute myeloid leukemia to retinoic acid (173). All this evidence and the enzymatic nature of PIN1 make it an attractive drug target.

Considerable effort for developing a PIN1 target therapy has been made, yet the provided inhibitors mainly display low potency, specificity, stability, and permeability [for comprehensive reviews about PIN1 inhibitors, see (7, 8, 174)]. Nonetheless, the use of liposomal formulations to vehicle a potent, but not cell-permeable, PIN1 inhibitor in cancer cells has shown effectiveness in curbing tumor growth *in vivo* (175), implying that such a strategy could be applied to other inhibitors with scarce pharmacokinetic properties.

We have recently discovered a PIN1 inhibitor with a peculiar mechanism of action. This new small molecule, KPT-6566, acts as a specific and covalent inhibitor of PIN1 that in addition causes its degradation. Intriguingly, from the KPT-6566-PIN1 binding reaction, a ROS-producing and DNA damaging molecule is released with exacerbated cytotoxic activity toward cancer cells. The robust tumor-killing activity observed both *in vitro* and *in vivo* very likely relies on the exhaustion of CSCs, ROS overload and impairment of anti-apoptotic pathways in bulk tumor cells (19).

All-trans retinoic acid (ATRA) and arsenic trioxide (ATO), recently discovered to inhibit PIN1 (166, 176), are the most attractive compounds to be rapidly repurposed as PIN1 inhibitors in clinical trials, being already FDA approved and showing efficacy against acute promyelocytic leukemia (APL) both alone and in combination. Still their usage poses challenges for an application against solid tumors, mainly because of the poor stability of ATRA (166, 176). Novel formulations of ATRA have now shown efficacy against liver cancers (177), paving the way toward an effective PIN1 therapy in humans.

Drug screenings with both synthetic and natural compound libraries have revealed several interesting hits that deserve further pharmacological development (7, 20, 178). Some of the drugs derived from natural sources have gained interest due to a reported effective PIN1 inhibition *in vitro* and in pre-clinical models, such as Juglone from Walnut tree or Epigallo-cathechin-gallate (EGCG) from green tea, even though they have toxic or non-specific activities against other cellular targets, respectively (7, 8, 174). Nevertheless, this prompted new efforts for finding PIN1 inhibitors by screening libraries of molecules derived from natural products (179, 180). Of special interest are also intracellular compounds, such as 4-Hydroxynonenal (HNE), a product arising from lipid oxidation, able to inhibit PIN1 by adducting Cysteine 113 within its catalytic pocket (181).

## Conclusions and Perspectives

Regulation of the phosphorylation and transcriptional landscapes by PIN1 establishes this enzyme as a central effector of dynamic molecular switches imposed by environmental and cell-intrinsic cues. But, in spite of a huge amount of data reporting a role for PIN1 on specific substrates and pathways, a comprehensive picture of its biological effects is still elusive. In particular, a still puzzling aspect of PIN1 regards its ability in conferring different and often opposite cell fates by operating concomitantly on a multitude of proteins. This is an important question, since deregulation of such molecular programs likely underlies cancer development, where tumor suppressors are inactivated while oncogenes simultaneously upregulated. In addition, not much is known about how PIN1 function is developmentally regulated *in vivo* under defined circumstances and which are the biological consequences for single cells, tissues, and the whole organism.

Studies performed in animal and human models have begun to shed light on these issues. Indeed, they indicate that PIN1 expression, activity, and subcellular localization are complex and regulated at multiple levels *in vivo*. Moreover, they demonstrate how particular stress signals impact PIN1 activity, which is in turn crucial in transducing adequate or pathogenic cellular responses through reprogramming of different molecular networks at several levels (Figure 1). It will be important to find out which are the clinical and therapeutic consequences of blocking such signal transduction or the downstream molecular responses.

Along this vein, it has been observed that PIN1 is overexpressed in the majority of human tumors and higher levels of PIN1 are generally associated with poorer prognoses (7). However, PIN1 does not work as an independent prognostic factor in most human cancers, except for prostate cancer (182). We might speculate that PIN1 association with prognosis very likely is still ill defined, since most studies were based on either RNA or protein levels, that do not necessarily correlate with its activity. Indeed, both activating and inactivating PTMs on PIN1 have been observed in different cancers that often also correlated with specific subcellular localizations (7, 8). It is thus conceivable that an appropriate evaluation of the PIN1 activation status in tumor tissues will be more predictive of its activity rather than solely total protein or mRNA levels. In addition, the importance of PIN1 in cancer development may depend on the levels of key oncogenic substrates, such as mutant p53 proteins, that act as prognostic markers in association with PIN1 in breast cancer (16).

Another important level of regulation regards the impact of nutritional cues, such as glucose and lipids, on PIN1 biology. Enhanced glucose levels in fact cause transcriptional de-repression of the mouse *Pin1* promoter, and upregulation of PIN1 protein levels that in turn spur ROS production and inflammation, highlighting possible novel mechanisms of PIN1 deregulation in pathogenic conditions such as obesity, diabetes, and cancer (48, 123). These insights obtained from studies in mouse models, coupled with the fact that PIN1 helps enforcing the Warburg effect at multiple levels, support the notion that one crucial developmental function of PIN1 is to integrate metabolic cues with cell proliferation, and that this is deregulated in cancer.

Such information will crucially impact future cancer prevention programs and therapies based on PIN1 inhibition. For instance, it will be important to assess the impact of the diet and life style on PIN1 also in humans, and to validate PIN1 as a potential biomarker and target for metabolic diseases such as diabetes (123) and cancer. In this context, also the nutritional status of neoplastic patients might be an important issue to consider since it is associated with tolerability to conventional therapies (183). Moreover, given the impact of PIN1 in p53-dependent apoptosis, PIN1 based therapies could underlie also radiation- and chemotherapy induced sickness in healthy tissues, especially in those with high PIN1 levels (6). It will thus be relevant to develop more specific and potent PIN1 inhibitors, and elaborate better combination therapies and strategies for drug delivery. In addition more sophisticated animal models are required to improve preclinical investigation, such as stage- and tissue-specific *Pin1* conditional *knock-out* mice (124), or human genetically modified (e.g., gene edited) organoids that could open new horizons for the clinical investigation of PIN1-based therapies with immediate translational potential (184).

## Author Contributions

AZ, AR, and GDS: conception and design and writing of the manuscript; EC: assisted in the preparation of the manuscript.

### Conflict of Interest Statement

The authors declare that the research was conducted in the absence of any commercial or financial relationships that could be construed as a potential conflict of interest.
